# Community-Based Hereditary Breast and Ovarian Cancer Family History Assessment and Genetic Testing among Spanish-Speaking Hispanic/Latina Women in California

**DOI:** 10.1158/2767-9764.CRC-26-0046

**Published:** 2026-09-10

**Authors:** Daisy M. Montoya, Amanda F. Corpuz, Karla E. Gonzalez, Kaylee Santos, Alyssa Reed, Julie H.T. Dang, Helen K. Chew, Guadalupe Polanco-Echeverry, Lizeth I. Tamayo, Miriam T. Hernandez, Alejandra Martinez, Elad Ziv, Susan L. Neuhausen, Luis G. Carvajal-Carmona, Ysabel Duron, Laura Fejerman

**Affiliations:** 1 University of California, Davis, Davis, California.; 2 Department of Public Health Sciences, University of Chicago, Chicago, Illinois.; 3 Visión y Compromiso, Los Angeles, California.; 4 Promoters for Better Health, Pomona, California.; 5 University of California, San Francisco, San Francisco, California.; 6 Department of Population Sciences, Beckman Research Institute of the City of Hope, Duarte, California.; 7 The Latino Cancer Institute, San Jose, California.

## Abstract

Breast cancer is the most common cancer among women in the United States and the leading cause of cancer incidence and mortality among Hispanic/Latina (H/L) women. For women with a genetic predisposition to hereditary breast and ovarian cancer (HBOC), genetic counseling has been shown to aid in the decision-making process and engagement in risk-reducing strategies for cancer prevention and early detection. However, H/L women are less likely to receive genetic testing and undergo genetic counseling than non-Hispanic White women. We developed a *promotores*-led virtual HBOC outreach, education, and risk assessment program [*Tu Historia Cuenta* (THC)] for Spanish-speaking H/L women in California. In this study, we describe genetic testing uptake, testing results, and participants’ experiences. Participants (*N* = 1,286) responded to a demographic and HBOC family history survey. Those identified as high risk based on the family history survey were offered free genetic testing with a hereditary cancer panel. Of the 111 women identified as high risk, 18 had been previously tested, and 33 received genetic testing through THC, resulting in a 35.5% completion rate for those offered testing. Among the 51 women with test results, 6 had a positive result, 3 were carriers of a variant of unknown significance, 38 had a negative result, and 4 participants tested before recruitment did not know their results. Participants who tested through THC indicated satisfaction with the genetic testing process. These findings underscore the importance of simplifying access and exploring additional factors influencing H/L women’s decision to receive testing beyond previously established cost, awareness, and knowledge barriers.

**Significance:**

Utilization of genetic testing and genetic counseling among H/L women in the United States remains limited. The *promotores*-led THC is one of the first community-based outreach programs for Spanish-speaking H/L women outside of a healthcare setting. Overcoming barriers such as language, cost, and knowledge, we identified additional obstacles such as testing kit activation and fear of future costs.

## Introduction

Breast cancer is the most common cancer among women in the United States (1) and the leading cause of cancer-related deaths among Hispanic/Latina (H/L) women (2). H/L women have a lower incidence rate of breast cancer compared with non-Hispanic White (NHW) women (3) but are more likely to be diagnosed at later stages (4) and have a higher risk of breast cancer–specific mortality (3). Compared with NHW, H/L women have a 164% higher risk of dying of breast cancer before the age of 50 years (5). Inequities in breast cancer among H/L women can be largely attributed to limited access to healthcare, including diagnostic follow-up, lower rates of mammography utilization, provider bias, and educational and language barriers (2, 6, 7). Such barriers are an added challenge to the access and use of timely preventive services (e.g., screening and early detection), treatment, and follow-up care (8).

Approximately 5% to 10% of breast cancer cases can be attributed to inherited genetic mutations in high-penetrance genes such as *BRCA1* and *BRCA2* (9). Women who carry these mutations face a 60% to 70% lifetime risk of developing breast cancer (10), underscoring the critical importance of genetic testing and genetic counseling for early detection, prevention, and risk management. H/L women are less likely to undergo genetic services than NHW women (11, 12), which is likely explained by their significantly lower genetic testing/genetic counseling awareness/knowledge levels, as well as barriers to access (10, 13).

Previous studies have demonstrated the potential to increase genetic testing/genetic counseling uptake for hereditary breast cancer risk assessment among H/L women. One study developed a genetic cancer risk assessment program for uninsured and underinsured, primarily H/L women, most of whom had a breast cancer diagnosis (72%; ref. 14). Of the 125 study participants who underwent genetic counseling risk assessment, 67% were offered genetic testing based on their risk, 96% of whom accepted *BRCA1/2* testing, achieving high uptake despite significant barriers (14). Several studies examining the knowledge and experiences of H/L women with hereditary breast and ovarian cancer (HBOC) genetic services noted that once informed, H/L women demonstrated positive attitudes and high interest in genetic services (15–17). When provided with access to genetic testing/genetic counseling services, H/L women were less hesitant to utilize these services; however, culturally and linguistically appropriate genetic testing/genetic counseling navigation programs for H/L women at risk of HBOC are limited, especially for the general population without a breast cancer diagnosis.

Community health educators, also known as *promotores* in the H/L community, are trusted community members who serve as liaisons between the H/L community and the healthcare system. They are uniquely positioned to bridge this gap by tailoring medical information to the community’s language, culture, and health literacy levels (18–21). *Promotores*-led educational interventions are cost-effective and have been shown to significantly increase breast cancer–related knowledge among participants (22–26).

In partnership with the Latino Cancer Institute, “*Tu Historia Cuenta*” (THC) was developed to address disparities in HBOC risk assessment and testing through culturally tailored outreach, education, and navigation for monolingual Spanish-speaking H/L women in three cities in California (Los Angeles, San Francisco, and Sacramento; refs. 8, 27). Using a *promotores*-led model, the program aimed to increase awareness and knowledge about HBOC, identify H/L women with strong family histories, provide genetic testing, and follow up with genetic counseling when needed. During earlier phases of the THC program, *promotores* were trained to deliver educational sessions (8) and administer a family history survey, which was used to identify high-risk participants based on their family or personal history of cancer, who were then offered genetic testing (27). THC previously reported on the participants’ characteristics (i.e., demographics, screening behavior, genetic testing knowledge), barriers to education, and feedback on the THC educational materials (8, 27–29). The development and implementation of THC were based on the construct of relational culture (30), adult learning theory (31, 32), and implementation with a continuous stakeholder engagement approach. Iterative modifications of materials and activities were informed by ongoing feedback, monitoring, and evaluation (8, 27–29). In this study, we present updated demographic characteristics and genetic risk screening uptake of program participants at the end of the implementation period. We also report the outcomes of the navigation process and experiences with, and results of, genetic testing for those identified as high risk.

This program uniquely positions *promotores* not only as trusted community leaders to conduct outreach and education about HBOC but also as integral facilitators in the implementation of the family history survey to identify high-risk participants and facilitate genetic testing. To our knowledge, this is among the first community-based, prevention-focused programs, outside a healthcare setting, to educate Spanish-speaking H/L women about HBOC, the majority of whom do not have a prior breast cancer diagnosis, and navigate those at risk of HBOC through genetic testing and genetic counseling.

## Materials and Methods

### Study design and population

THC is a real-world implementation of a community-based, *promotores*-led initiative focused on HBOC education, risk identification, and facilitation of genetic testing and counseling in Northern and Southern California. Spanish-speaking *promotores* residing in the cities of Los Angeles, San Francisco, and Sacramento leveraged their community networks to recruit H/L women for remote or in-person HBOC education sessions and facilitated access to genetic testing. Recruitment began in June 2020 and was led by two community–based organizations in Northern and Southern California. Eligible participants were self-identified Spanish-speaking or bilingual H/L women between the ages of 21 and 75. *Promotores* conducted outreach to the community broadly, advertising their classes using multiple channels, including social media, school mailers, and word of mouth. Because of this real-world *promotores*-driven approach, we could not assess how many of the individuals who learned about the program decided not to participate. However, if people filled out the demographic questionnaire, we were able to measure what percentage attended the education session, as the family history survey was shared at the end of the class. Some participants attended the education session and filled out the family history survey without having filled out the demographic survey, but that group was a minority. Participation in the program was indicated by completing at least one survey, but for the current analysis, both the demographic and family history surveys had to be completed.

By the end of the recruitment period in December 2023, 1,700 H/L women in California had registered for the THC education sessions and completed the demographic survey or the HBOC family history survey. Participants provided verbal informed consent to participate in surveys and education sessions and written informed consent for participating in genetic testing and counseling. This study was approved by the UC Davis Institutional Review Board.

### Program description

THC is a *promotores*-led outreach and education program that utilized culturally tailored materials developed through a continuous stakeholder engagement approach (8). Hour-long educational sessions were conducted to provide participants with foundational knowledge about breast cancer, with a particular focus on hereditary breast cancer and genetics (8).

Three surveys were administered to program participants using a phone-based Qualtrics link during the education and risk assessment phase: a demographic survey, a HBOC family history survey, and a feedback survey, all of which have been described elsewhere (8, 29). Briefly, the HBOC family history survey was adapted from the referral screening tool (33), which has been previously validated and is listed by the United States Preventive Services Task Force as a tool that can be used for genetic counseling referral. Each “yes” response on the survey had an associated score of 2, 4, or 6, depending on the age of onset and type of cancer reported for self and family members. Participants with a score of 6 or higher were considered to have responded in a manner consistent with a strong family history of breast/ovarian cancer. For THC, we broadened the criteria for testing to include women with a breast cancer diagnosis at any age. The THC program offered free genetic testing to any participant with a score of 6 or higher or who had been diagnosed with breast cancer.

Two paper-based validated surveys were administered after genetic testing and counseling: the Decision Regret Scale and the Feelings About genomiC Testing Results (FACToR) questionnaires (34, 35). The Spanish version of the Decision Regret Scale is a reliable tool to evaluate regret (ω = 0.87; ref. 36). The FACToR questionnaire had previously been translated into Spanish and used in a study comparing the delivery of genetic test results through letters versus telephone (37). Although psychometric validation of a Spanish-language version of the FACToR questionnaire is limited, its prior use demonstrates appropriateness for use in Spanish-speaking populations. Two of the twelve questions from the FACToR questionnaire were removed due to their lack of applicability to the study.

Genetic testing was offered to participants through a collaboration with a direct-to-consumer testing company that offers clinical-grade genetic testing. We used Color Genomics’ Hereditary Cancer Panel, which screens for mutations in 29 genes (*APC*, *ATM*, *BAP1*, *BARD1*, *BMPR1A*, *BRCA1*, *BRCA2*, *BRIP1*, *CDH1*, *CDK4*, *CDKN2A*, *CHEK2*, *EPCAM*, *GREM1*, *MITF*, *MLH1*, *MSH2*, *MSH6*, *MUTYH*, *PALB2*, *PMS2*, *POLD1*, *POLE*, *PTEN*, *RAD51C*, *RAD51D*, *SMAD4*, *STK11*, and *TP53*). Genetic tests were ordered for participants who were identified as high risk based on the cancer family history survey and who consented to genetic testing by a UC Davis breast medical oncologist. Saliva kits were shipped directly to participants by Color Genomics. In addition to Color Genomics’ instructional brochure on how to provide the saliva sample, participants received direct navigation from the *promotores* on how to activate the kits.

Test results were reviewed by a board-certified Spanish-speaking genetic counselor at the UC Davis Comprehensive Cancer Center Hereditary Cancer Genetics Program. Participants with negative results received their reports directly, whereas those with positive or uncertain findings were contacted to schedule a genetic counseling session. Regardless of the results, all participants were followed up through Zoom or phone call by the genetic counselor to ensure they understood their results, the implications for future breast cancer risk, and the recommended screening timeline.

### Data processing and analysis

The *promotores* collaborated closely with the research team to verify individual risk scores and confirm risk status. Participants identified as high risk based on family and personal history of breast cancer were invited to undergo genetic testing. Lastly, all participants were provided with a post–genetic testing survey, 2 months after receipt of their genetic test report, to assess their feelings and possible regrets about completing the process. The post–genetic testing survey was distributed by the *promotores* or the genetic counselor, depending on the need for genetic counseling.

All data were updated in a master tracking sheet, and if any discrepancies arose, the *promotores* were directly contacted for clarification from the participant. The master tracking sheet was crucial in ensuring participant retention by verifying that they completed every part of the THC genetic testing process. The tracking sheet included a “notes” section in which the *promotores* wrote in free text format a description of the outcome of their outreach efforts for individuals flagged as high risk by the cancer family history survey. These “notes” were used to provide insights about reasons given by participants who declined the offer of genetic testing.

Descriptive statistics are reported as means with standard deviation (SD) for continuous variables and as proportions for categorical variables. We conducted comparative analyses to examine demographic differences between high- and non–high-risk groups, as well as among four follow-up status categories within the high-risk group: previously tested, tested through THC, lost to follow-up, and declined. For continuous variables, *P* values were calculated using two-sided *t* tests or analysis of variance. For categorical variables, associations with risk status or follow-up category were assessed using the Fisher exact test or *χ*^2^ test, as appropriate. All analyses were performed using RStudio (RRID: SCR_000432; ref. 38).

### Quality control processes

A total of 1,700 unique participants completed the demographic survey and/or the HBOC family history survey. Because family history data were essential for determining risk status, individuals who completed only the demographic survey were excluded from the analyses. Following quality control procedures (i.e., removal of duplicate entries and those without full information for risk assessment), 1,286 HBOC family history survey responses were retained, forming the final analytic sample for this study. Of those, 1,228 participants also completed the demographic survey. As 58 participants completed the family history survey but not the demographics survey, they appear as “missing” in the analysis of demographic variables. The group of 406 participants who completed the demographic survey but not the family history survey (excluded from the current analysis) had similar demographic characteristics to the group of 1,286 individuals who completed the family history survey, except for the distribution of insurance type; the group of participants who had only completed the demographic survey included a smaller proportion of individuals without insurance and a larger proportion of individuals with public insurance (Supplementary Fig. S1; Supplementary Table S1).

## Results

### Overall participants’ demographic characteristics

The average age of the 1,286 participants included in analyses was 42.8 years (SD = 10.5). On average, participants had lived in the United States for 18.9 years (SD = 10.7; Table 1). Educational attainment varied: ∼6% of participants reported no formal education, 20.3% reported completion of elementary school, 15.9% reported completion of middle school, 30.3% reported completion of high school, 12.4% reported having an associate degree, and 9.8% reported having a university degree. The program included women with varying degrees of English fluency; 18.4% were monolingual Spanish speakers, 33.2% had limited English proficiency, 29.1% were conversational in English, and 14.4% were fully bilingual. With respect to health insurance, 46.7% had public health insurance, 34.8% were uninsured, and 13.2% had private insurance. The average household size was 4.4 individuals (SD = 1.8).

**Table 1.** Demographic characteristics of THC participants overall and by risk groups (high-risk/non–high-risk).

Population demographic, mean (SD) or *n* (%)				
Characteristic	Overall (*N* = 1,286)	Non–high-risk (*N* = 1,175)	High-risk (*N* = 111)	*P* value
Age	42.8 (10.5)	42.6 (10.5)	45.7 (9.5)	0.0013
Years residing in the United States	18.9 (10.7)	18.7 (10.7)	21.8 (10.6)	0.0046
English language proficiency				
Monolingual Spanish speaker	237 (18.4)	224 (19.1)	13 (11.2)	0.028
Limited English use	427 (33.2)	395 (33.6)	32 (28.8)	​
Conversational	374 (29.1)	329 (28)	45 (40.5)	​
Bilingual	185 (14.4)	168 (14.3)	17 (15.3)	​
Missing	63 (4.9)	59 (5)	4 (3.6)	​
Health insurance				
No insurance	448 (34.8)	416 (35.4)	33 (28.8)	0.061
Public	601 (46.7)	549 (46.7)	52 (46.9)	​
Private	170 (13.2)	147 (12.5)	23 (20.7)	​
Missing	67 (5.2)	63 (5.4)	4 (3.6)	​
Educational attainment				
No school	75 (5.8)	71 (6)	4 (3.6)	0.044
Elementary school	261 (20.3)	241 (20.5)	20 (18)	​
Middle school	204 (15.9)	186 (15.8)	18 (16.2)	​
High school	390 (30.3)	362 (30.8)	28 (25.2)	​
Associate degree	159 (12.4)	142 (12.1)	17 (15.3)	​
University	126 (9.8)	106 (9)	20 (18)	​
Missing	71 (5.5)	67 (5.7)	4 (3.6)	​
Number of people in household	4.4 (1.8)	4.40 (1.8)	4.1 (1.6)	0.043

### Demographic characteristics by risk group

A total of 1,286 women completed the family history survey. Based on the calculated risk score from these responses, participants were categorized as either non–high-risk or high-risk groups for HBOC. The average age of participants differed significantly between the non–high-risk and high-risk groups. Participants in the high-risk group were about 3 years older [average (AVG), 45.7 vs. 42.6; *P* value = 0.0013] and, on average, had resided 3 years longer in the United States (AVG, 21.8 vs. 18.7; *P* value = 0.0046) compared with those in the non–high-risk group (Table 1). Differences in English language proficiency and educational attainment between high-risk and non–high-risk groups were statistically significant (*P* value = 0.028 and 0.044, respectively). The non–high-risk group included a larger proportion of participants with less than limited English use and who had attained a high school level education or less. In contrast, the high-risk group had a greater proportion of participants with at least conversational English language proficiency and an associate’s degree or higher. Participants in the non–high-risk group were more likely to report having no health insurance (35.4%) or public insurance (46.7%). On average, participants in the non–high-risk group lived in larger households (4.4) compared with those in the high-risk group (4.1).

### Demographic characteristics of women in the high-risk category by testing status

On average, participants who had previously undergone genetic testing were older (48.2 years) and had lived in the United States for a shorter period (21.1 years) compared with other high-risk participants (Table 2). In comparison, those tested through THC were younger (44.2 years) and had resided in the United States longer (22.9 years). Participants in the high-risk group tested through THC also had the largest proportion of at least conversational English proficiency, whereas those previously tested were more likely to report limited English proficiency or less. The relationship between testing status and health insurance coverage was statistically significant (*P* value = 0.0087). High-risk category individuals who declined genetic testing were the most likely to be uninsured (40.5%), previously tested participants were more likely to have public insurance (61.1%), and those tested through THC had the largest proportion of private health insurance (39.4%). Previously tested participants were more likely to have attained a middle school education or less, whereas participants tested through THC were more likely to have postsecondary education. Additionally, participants who declined testing lived in larger households (AVG, 4.3 individuals) compared with the other groups (Table 2).

**Table 2.** Demographic characteristics of 111 women in the high-risk category based on the HBOC family history survey by genetic testing status.

Demographic of high-risk population (*N* = 111) by testing status, mean (SD) or *n* (%)					
Characteristic	Previously tested (*N* = 18)	Tested through THC (*N* = 33)	Lost to follow-up (*N* = 23)	Declined (*N* = 37)	*P* value
Age	48.2 (10.1)	44.2 (9.2)	45.5 (10)	45.9 (9.2)	0.54
Years residing in the United States	21.1 (9.5)	22.9 (12)	21.9 (10.4)	21 (10.3)	0.89
English language proficiency					
Monolingual Spanish speaker	3 (16.2)	2 (6.1)	3 (12)	5 (13.5)	0.22^a^
Limited English use	8 (44.4)	7 (21.2)	4 (17.4)	13 (35.1)	​
Conversational	7 (38.9)	15 (45.5)	10 (23.5)	13 (35.1)	​
Bilingual	0 (0)	8 (24.2)	5 (21.8)	4 (10.8)	​
Missing	0 (0)	1 (3)	1 (4.4)	2 (5.4)	​
Health insurance					
No insurance	7 (38.9)	6 (18.9)	4 (17.4)	15 (40.5)	0.0087^a^
Public	11 (61.1)	13 (39.4)	13 (56.5)	15 (40.5)	​
Private	0 (0)	13 (39.4)	5 (21.7)	5 (13.5)	​
Missing	0 (0)	1 (3)	1 (4.4)	2 (5.4)	​
Educational attainment					
No school, elementary school, or middle school	10 (55.6)	10 (30.3)	7 (30.4)	15 (40.5)	0.52
High school	4 (22.2)	7 (21.2)	7 (30.4)	10 (27)	​
Postsecondary education	4 (22.2)	15 (45.5)	8 (34.8)	10 (27)	​
Missing	0 (0)	1 (3)	1 (4.4)	2 (5.4)	​
Number of people in household	4.1 (1.9)	4 (1.3)	4 (1.4)	4.3 (1.7)	0.86

a *P* values for English language proficiency and health insurance should be interpreted cautiously due to low cell counts in the use of the Fisher exact test.

### Genetic testing navigation and results

Among the 1,286 participants who completed the HBOC family history survey, 111 women (8.6%) were identified as high risk (Table 3). Women identified as high risk were offered free genetic testing and counseling. Of the high-risk group, 37 (33.3%) declined testing (Table 3), 33 (29.7%) received and successfully used a genetic testing kit, 23 (20.7%) were lost to follow-up, and 18 (16.2%) had previously undergone genetic testing. Participants were considered lost to follow-up either because they could not be reached after multiple attempts by *promotores* (“unresponsive,” 18/23, 78.3%) or due to difficulties activating their kits, even with support from *promotores* (“activation issues,” 4/23, 17.4%). One participant died during the study period. Among the 51 participants who completed a genetic test, 6 (11.8%) received a positive result, 38 (74.5%) tested negative, and 3 (5.9%) had variants of uncertain significance (VUS; Table 3). The uptake of genetic testing among those who had not been previously tested was 35.5% (Table 3).

**Table 3.** Completion rates for the different components of THC and participants’ genetic testing results.

THC component	*n* (%)
HBOC family history survey (*N* = 1,286)	
Self-reported breast cancer survivor	31 (2.4)
Identified as high risk	111 (8.6)
Follow-up of high risk (*N* = 111)	
Previously tested	18 (16.2)
Lost to follow-up	23 (20.7)
Tested through THC	33 (29.7)
Declined	37 (33.3)
Testing uptake if not previously tested	33/93 (35.5)
Reason for lost to follow-up (*N* = 23)	
Unresponsive	18 (78.3)
Activation issue	4 (17.4)
Deceased	1 (4.4)
Results (includes tested previously and tested through THC; *N* = 51)	
Positive^a^	6 (11.8)
Negative	38 (74.5)
VUS	3 (5.9)
Unknown	4 (2)

a One woman tested through THC received a positive result and had a pathogenic variant in the *BRCA1* gene. The other five positive results were reported by participants who had been tested before joining the THC education session.

Weekly communication documents between the research team and *promotores* included a section in which the *promotores* wrote free notes about reasons given by participants for declining the invitation to genetic testing. Of the 37 participants who declined testing, 20 (54%) did not provide additional information to the *promotores*. A total of seven individuals (19%) explained that they preferred to handle the testing with their own doctors and did not want to continue to be part of the THC testing process. The remaining individuals expressed concerns about clinic barriers and insurance/cost issues if tests were positive, fear of results, or mentioned their busy work schedules (Table 4).

**Table 4.** Reasons provided to *promotores* for declining genetic testing.

Reason for declining testing (*N* = 37)	*n*	Percentage
Declined, no further explanation	20	54%
Left the United States	3	8%
Concerned about clinic barriers and cost	2	5%
Busy with work	2	5%
Concerned about no insurance	1	3%
Preferred self-navigation	7	19%
Fear of test results and other health concerns	1	3%
Did not want to provide date of birth	1	3%

### Post–genetic testing results surveys (FACToR questionnaire and Decision Regret Scale)

Of the 33 participants who were successfully navigated to genetic testing through THC, 23 (69.7%) completed both the “FACToR” questionnaire and the “Decision Regret Scale” survey after receiving their results. Among these 23 participants who completed both surveys, 1 (4.3%) received a positive result, 21 (91.3%) received a negative result, and 1 (4.3%) received a VUS result.

When asked how upset or sad they felt about their genetic test results, 21 participants (91%) reported not feeling upset at all, and 20 (87%) responded not feeling sad at all (Table 4). In contrast, when asked about positive emotions, 22 participants (96%) reported feeling “a good deal” or “a great deal” of happiness, and all 23 (100%) reported feeling similarly relieved. With respect to their understanding of the results, 17 participants (74%) indicated feeling “a little” or “not at all” unsure, whereas 6 (26%) indicated feeling “somewhat” unsure. Similarly, participants were more likely to feel “somewhat” to “not at all” uncertain about what their results mean for their children’s/family’s disease risk. A majority indicated feeling “good” or “great” about understanding their options for disease prevention or early detection although eight participants (34%) reported feeling “somewhat” to “not at all” informed. Participants were most likely to feel “somewhat” to “not at all” concerned about how their results would affect their health insurance status. All participants indicated their results to be at least “somewhat” or more helpful in planning for the future (Table 5).

**Table 5.** FACToR questionnaire and results.

FACToR questionnaire (*N* = 23); *n* (%)	Not at all	A little	Somewhat	A good deal	A great deal
How upset did you feel about your genetic test result?	21 (91)	2 (9)	0 (0)	0 (0)	0 (0)
How happy did you feel about your genetic test result?	0 (0)	0 (0)	1 (4)	8 (35)	14 (61)
How anxious or nervous did you feel about your genetic test result?	8 (35)	4 (17)	7 (30)	3 (13)	1 (4)
How relieved did you feel about your genetic test result?	0 (0)	0 (0)	0 (0)	10 (43)	13 (57)
How sad did you feel about your genetic test result?	20 (87)	2 (9)	1 (4)	0 (0)	0 (0)
How unsure did you feel about what your genetic test result means to you?	13 (57)	4 (17)	6 (26)	0 (0)	0 (0)
How uncertain did you feel about what your genetic test result means for your child(ren) and/or family’s disease risk?	4 (17)	7 (30)	9 (39)	1 (4)	2 (9)
How much did you feel you clearly understood your options for disease prevention or early detection?	1 (4)	4 (17)	3 (13)	10 (43)	5 (22)
How concerned were you that your genetic test result would affect your health insurance status?	9 (39)	7 (30)	2 (9)	3 (13)	2 (9)
How helpful was the information you received from your genetic test results in planning for the future?	0 (0)	0 (0)	3 (13)	8 (35)	12 (52)

When asked about participants’ regret about completing the genetic testing, 100% of respondents agreed or strongly agreed they had made the right decision, would choose to take the test again, and considered their decision to be a wise one (Table 6). Nearly all participants (22, 96%) disagreed or strongly disagreed with statements indicating regret or feeling their decision caused them harm. Only one participant agreed with both statements (Table 6). All respondents, regardless of their genetic test results (negative, positive, or VUS), responded similarly to both surveys.

**Table 6.** Genetic testing Decision Regret Scale survey and results.

Genetic testing Decision Regret Scale (*N* = 23); *n* (%)	Strongly agree	Agree	Neither agree nor disagree	Disagree	Strongly disagree
It was the right decision.^a^	19 (86)	3 (14)	0 (0)	0 (0)	0 (0)
I regret the choice that was made.	0 (0)	1 (5)	0 (0)	14 (61)	8 (35)
I would go for the same choice if I had to do it over again.	19 (83)	4 (21)	0 (0)	0 (0)	0 (0)
The choice did me a lot of harm.	0 (0)	1 (5)	0 (0)	5 (22)	17 (74)
The decision was a wise one.	19 (83)	4 (21)	0 (0)	0 (0)	0 (0)

a One response missing (*n* = 22).

## Discussion

The goal of this study was to describe the uptake and results of genetic testing among Spanish-speaking H/L women in California who could benefit from HBOC genetic testing and counseling and to provide insights into navigation to and experience with genetic testing. Through this program, we identified 111 women (8.6%) in the high-risk category based on a validated HBOC family history survey (33), 18 (16.2%) of whom had genetic testing for HBOC before joining the THC study. A total of 33 participants (29.7%) received genetic testing as part of the THC program. Among all THC study participants with a high-risk family history profile, six were identified as carriers of pathogenic mutations either through previous testing (5) or THC testing (1). THC identified three participants carrying a VUS (two of them from prior testing). Demographic factors associated with cancer screening behavior, genetic testing knowledge, and strong family history have been described in a prior article (27).

Overall, participants in the HBOC family history survey-based high-risk category had higher levels of education, were more likely to have private health insurance, and were more likely to have conversational levels of English proficiency or be fully bilingual compared with women in the non–high-risk category (Table 1). The family history survey used to assess participants’ risk is based on the participants’ knowledge of their family history of cancer. Women with higher levels of education and socioeconomic status have higher levels of health literacy than those with lower levels (39), which could lead to more complete knowledge about the family history of cancer. This could explain the overrepresentation of women with lower socioeconomic status and education in the non–high-risk category, highlighting the need for additional education in low-health literacy communities about the importance of sharing health history, including cancer history, with family members.

A total of 33 women (29.7%) in the high-risk category were successfully navigated into genetic testing by THC, and an additional 18 (16.2%) had previously undergone testing, representing an overall testing rate of 45.9% (51/111) among high-risk participants and a testing rate of 35.5% (33/93) for the THC program (Table 3). As part of the statewide Georgia Center for Oncology Research and Education’s family history screening program, 264 eligible H/L women were offered no-/low-cost genetic testing and counseling within clinic settings. Of the eligible women, 107 opted to receive genetic counseling (40.5%), and of those, 103 completed genetic testing, resulting in a 39% testing rate (40). This level of uptake was attributed to language barriers and low genetic literacy in the Georgia study. However, THC provided HBOC and genetic testing education in Spanish to all participants prior to being offered the genetic testing services and still encountered a 20.7% “loss to follow-up” and a 33.3% “declined to participate in genetic testing” rate, suggesting that other factors might be important (e.g., emotional barriers or competing health concerns of higher priority for participants).

Previous studies have reported that if given the opportunity, proper education, and access, H/L women express interest in genetic testing uptake (15–17). However, despite the removal of the financial cost for testing and the need for in-person visits or travel to access genetic testing and counseling among H/L women in the THC study, 54.1% of participants were either lost to follow-up or declined to consent to genetic testing. More research is needed to explore other contributing factors, but based on notes taken by *promotores* after THC participants declined testing, H/L women’s genetic testing uptake may be influenced by concerns about potential future costs beyond the cost of the test, clinic barriers to timely care, preference for self-navigation, lack of time due to busy work schedules, and fear of results. Half of the people who declined testing (54%) did not provide further explanation (Table 4). Family impact and adverse emotional responses have been identified as barriers to genetic testing among H/L women for skin cancer (41). In a study conducted with at-risk H/L women about *BRCA* genetic counseling, emotional concerns (i.e., fear, distress) and competing life concerns (i.e., low priority, caregiving, family obligations, busy schedules) were identified as perceived barriers to genetic counseling (42). Consistent with this finding, a qualitative study that identified influences on the decision-making process about genetic testing and genetic counseling for breast cancer risk also found that H/L women prioritized family obligations over personal health needs (43). Addressing immediate costs, insurance, referrals, and language barriers alone is not enough to promote genetic testing uptake among H/L women. Concerns about emotional distress, family implications, and family obligations are barriers to genetic testing that have been previously reported among H/L women in the United States (41–43). Based on notes provided by the THC *promotores* about participants’ rationale for declining the offer of free genetic testing, an important concern was the future cost of healthcare and healthcare access barriers if test results were positive, highlighting the need for further research to develop effective strategies to address them.

Of the participants who consented to and completed testing, the majority responded positively to learning about their results (Table 5) and about their decision to have been tested, with nearly no participant regretting their decision (Table 6). All respondents, regardless of their genetic test results (negative, positive, and VUS), responded similarly to both surveys. Participants’ overall positive experience with genetic testing underscores the importance of further encouraging genetic testing services among H/L participants and addressing pretesting concerns about harm and emotional distress.

THC addressed barriers such as limited English proficiency, lack of educational knowledge, lack of genetic testing and genetic counseling awareness, lack of health insurance, and financial constraints through its structured educational and navigation process, leveraging Spanish-speaking *promotores* to educate H/L women on HBOC and to support high-risk participants in the uptake of genetic services at no cost to them. The flexibility of the genetic counselor is an added strength of this study. The genetic counselor was not constrained by a daily work schedule (calling mornings, evenings, and weekends) and spent a longer time talking with participants compared with typical genetic counseling visit durations.

Results should be interpreted with caution due to some limitations. Participant recruitment was conducted through the social networks of two *promotores* organizations located in the cities of Los Angeles, San Francisco, and Sacramento, which may limit the generalizability of the findings. THC materials were developed for California’s diverse H/L populations (8), and the generalizability of the program to other regions of the United States is not known. Furthermore, THC participants are not representative of the general H/L population of California, as THC sought to enroll women who were monolingual Spanish speakers or had limited English proficiency. Additionally, the family history survey used to assess cancer risk relied on participants’ self-reported knowledge, which could have led to misclassification, particularly underestimating the number of individuals at higher risk. THC’s main goal was to assess the uptake of genetic testing among H/L women if the process was simplified, led by educators from the community, and language, transportation, and cost barriers were removed. Hence, it is not a program implemented within the current health system workflow for HBOC risk assessment, counseling, and testing. It is also important to note that the program was explicitly promoted as HBOC education, likely attracting individuals with a personal or familial interest in HBOC, thereby introducing potential selection bias. Finally, educational materials, consent forms, Promotor navigation, and genetic counseling were all provided in Spanish. However, some of the materials in Color Genomics’ online interface were in English and not translated into Spanish, which required support from *promotores* for participants to navigate the website.

THC removed immediate transportation and cost barriers to genetic testing and counseling and provided education and navigation support in Spanish, contributing to efforts aimed at understanding how to mitigate HBOC testing disparities in the H/L community. Overall, participants who were fully navigated through genetic testing and counseling responded positively to the genetic testing process. However, 21% of the participants who were offered genetic testing did not respond to calls, and 33% explicitly declined the offer, highlighting the need to address barriers beyond language, education, transportation, and cost of testing. Future research on navigating H/L women to genetic testing should address concerns about potential psychologic harm, competing life stressors, and fear of future costs associated with positive genetic test results.

## Supplementary Material

Figure S1 Flow chart that describes the analytical sample.

Table S1 Table with the demographic characteristics of the study population (N=1692), by completion of the Family History Survey

## Data Availability

The raw, deidentified data supporting the conclusions of this article will be made available upon reasonable request by the authors.
