# Supplementary material for: Community-Based Hereditary Breast and Ovarian Cancer Family History Assessment and Genetic Testing among Spanish-Speaking Hispanic/Latina Women in California
Source: Cancer Res Commun. 2026 Sep 10;6(9):2126–35. doi: 10.1158/2767-9764.CRC-26-0046 (PMC13561150; doi:10.1158/2767-9764.CRC-26-0046)
Supplement: Supplementary file 1 [file crc-26-0046_figure_s1_suppsf1.doc]

**Figure S1: Schema of Criteria for Analytic Sample**
