# Supplementary material for: Community-Based Hereditary Breast and Ovarian Cancer Family History Assessment and Genetic Testing among Spanish-Speaking Hispanic/Latina Women in California
Source: Cancer Res Commun. 2026 Sep 10;6(9):2126–35. doi: 10.1158/2767-9764.CRC-26-0046 (PMC13561150; doi:10.1158/2767-9764.CRC-26-0046)
Supplement: Supplementary file 2 [file crc-26-0046_table_s1_suppst1.doc]

**Table S1: Demographics of Study Population (N=1692)* by Completion of Family History Survey**

|  | **Did not complete FH survey (N=406)** | **Completed FH survey (N=1286)**** | **p-value** |
| --- | --- | --- | --- |
| **Age, mean(sd)** | 42.5(10.4) | 42.82(10.48) | 0.55 |
| **Years in US, mean(sd)** | 18.1(11.2) | 18.92(10.68) | 0.18 |
| **Household size, mean(sd)** | 4.26(3.07) | 4.37(1.82) | 0.48 |
| **Insurance type, n(%)** | | | |
| None | 100(24.63) | 448(34.84) | <0.0001 |
| Private | 54(13.30) | 170(13.22) |  |
| Public | 245(60.34) | 601(46.73) |  |
| Missing | 7(1.72) | 67(5.21) |  |
| **English proficiency, n(%)** | | | |
| Monolingual Spanish | 59(14.53) | 237(18.43) | 0.11 |
| Limited English | 146(35.96) | 427(33.20) |  |
| Conversational | 120(29.56) | 374(29.08) |  |
| Fully bilingual | 74(18.23) | 185(14.39) |  |
| Missing | 7(1.72) | 63(4.90) |  |
| **Schooling, n(%)** | | | |
| None | 13(3.20) | 75(5.83) | 0.06 |
| Elementary | 72(17.73) | 261(20.30) |  |
| Middle School | 76(18.71) | 204(15.86) |  |
| High School | 127(31.28) | 390(30.33) |  |
| Associate’s degree | 68(16.75) | 159(12.36) |  |
| University | 39(9.61) | 126(9.80) |  |
| Missing | 11(2.71) | 71(5.52) |  |

*Excludes 8 individuals that completed the FH survey, but did not verify their score

**Includes 58 individuals who completed the FH survey, but not the demographic survey
